# Melatonin Enhances Heat Tolerance via Increasing Antioxidant Enzyme Activities and Osmotic Regulatory Substances by Upregulating *zmeno1* Expression in Maize (*Zea mays* L.)

**DOI:** 10.3390/antiox13091144

**Published:** 2024-09-22

**Authors:** Liru Cao, Abbas Muhammad Fahim, Xiaohan Liang, Senmiao Fan, Yinghui Song, Huafeng Liu, Feiyu Ye, Chenchen Ma, Dongling Zhang, Xiaomin Lu

**Affiliations:** 1 The Shennong Laboratory, Grain Crops Research Institute, Henan Academy of Agricultural Sciences, Zhengzhou 450002, China; caoliru008@126.com (L.C.); yinyutianqi2020@163.com (X.L.); fsmtmu@163.com (S.F.); yinghui820@163.com (Y.S.); lhfeng9110@163.com (H.L.); 18838971380@163.com (F.Y.); mchenchen1017@163.com (C.M.); zhangdongling0626@163.com (D.Z.); 2 College of Agronomy, Gansu Agricultural University, Lanzhou 730070, China; fahimabbaskhan@yahoo.com

**Keywords:** maize, heat stress, melatonin, enolase, antioxidant enzyme, osmoregulatory substances

## Abstract

Heat stress severely affects the yield and quality of maize. Melatonin (N-acetyl-5-methoxy-tryptamin, MT) plays an important role in various types of stress resistance in plants, including heat tolerance. Enolase (ENO, 2-phospho-D-glycerate hydrolyase) contributes to plant growth, development, and stress response. As of now, the molecular mechanisms by which MT and ENO1 affect heat tolerance are unknown. In our research, we have revealed that heat stress (H) and heat stress + MT (MH) treatment upregulate *ZmENO1* expression levels by 15 and 20 times, respectively. *ZmENO1* overexpression and mutant maize lines were created by transgenic and genome editing. These results illustrate that heat stress has a significant impact on the growth of maize at the seedling stage. However, ZmENO1-OE lines showed a lower degree of susceptibility to heat stress, whereas the mutant exhibited the most severe effects. Under heat stress, exogenous application of MT improves heat resistance in maize. The ZmENO1-OE lines exhibited the best growth and highest survival rate, while the *zmeno1* mutants showed the least desirable results. Following treatment with H and MH, the level of MT in ZmENO1-OE lines exhibited the greatest increase and reached the maximum value, whereas the level of MT in the *zmeno1* mutant was the lowest. Heat stress decreased the maize’s relative water content and fresh weight, although ZmENO1-OE lines had the highest and *zmeno1* mutants had the lowest. Heat stress led to an increase in the levels of MDA, hydrogen peroxide, and superoxide in all plants. Additionally, the ionic permeability and osmotic potential of the plants were significantly increased. However, the levels of MT were decreased in all plants, with the greatest decrease observed in the ZmENO1-OE lines. Interestingly, the *zmeno1* mutant plants had the highest expression levels of MT. Heat stress-induced upregulation of *ZmSOD*, *ZmPOD*, *ZmAPX*, *ZmCAT*, *ZmP5CS*, and *ZmProDH* in all plants. However, the ZmENO1-OE lines exhibited the greatest increase in expression levels, while the *zmeno1* mutants showed the lowest increase following MT spraying. The patterns of SOD, POD, APX, and CAT enzyme activity, as well as proline and soluble protein content, aligned with the variations in the expression levels of these genes. Our findings indicate that MT can upregulate the expression of the *ZmENO1 gene*. Upregulating the *ZmENO1 gene* resulted in elevated expression levels of *ZmSOD*, *ZmPOD*, *ZmAPX*, *ZmCAT*, *ZmP5CS,* and *ZmProDH*. This led to increased activity of antioxidant enzymes and higher levels of osmoregulatory substances. Consequently, it mitigated the cell membrane damage caused by heat stress and ultimately improved the heat resistance of maize. The results of this study provide genetic resources for molecular design breeding and lay a solid foundation for further exploring the molecular mechanism of MT regulation of heat stress tolerance in maize.

## 1. Introduction

Maize (*Zea mays* L.) is an important food and feed crop worldwide [1]. Ensuring a high and stable yield of maize plays an important role in ensuring food security and revenue growth. Globally, agricultural productivity is being negatively impacted by climate change. Heat stress is a significant concern, as it can swiftly and severely harm plant systems [2]. In 2022, eastern China experienced an unprecedented and intense heatwave during the crucial growth and development phase of maize [3]. This heatwave was characterized by exceptional heat stress, surpassing any recorded in the last 60 years. It lasted for an extended period and exhibited tremendous intensity [3]. Over the past few years, there has been an increase in the occurrence of severe weather conditions characterized by heat stress and drought. This has now become the usual pattern and poses a significant threat to maize production at both the seedling and harvest stages. Cultivating heat-stress-resistant cultivars is a crucial method for addressing this issue. Therefore, it is imperative to create heat-tolerant cultivars by exploiting heat-tolerant genes and employing molecular breeding technologies. Simultaneously, the utilization of plant hormones to enhance adaptation and safeguard plants against unfavorable climatic conditions is also considered one of the novel approaches [4]. Recently, there has been significant interest in melatonin (N-acetyl-5-methoxy-tryptamin), a hormone known for its potent physiological effects.

Melatonin (MT) is a low molecular weight indole compound that was first extracted from the pineal gland of cattle in 1958 [5]. Since then, an increasing number of studies have found that melatonin is also widely present in plants and microorganisms [6]. As an indole compound, MT can promote plant growth [5] and increase seed germination rates [7]. Furthermore, MT, which functions as a potent growth regulator and antioxidant, also has a significant impact on enhancing plant resilience to abiotic stressors [8]. It can improve stress tolerance in plants through both direct pathways, such as the direct removal of reactive oxygen species (ROS), and indirect pathways, such as increased antioxidant enzyme activity, photosynthetic efficiency, and metabolite content. Zhang Mingcong et al. [9] demonstrated that the regulatory impact of melatonin irrigation on soybean yield in unfavorable conditions surpassed that of foliar spraying. Through extensive research, it has been discovered that under normal physiological conditions, the concentration of melatonin in plants is typically quite low, ranging from pmol/g fresh weight to nmol/g fresh weight [10]. However, when plants are exposed to stressors such as heat stress, low temperature, drought, salt, heavy metals, ultraviolet radiation, or waterlogging, the concentration of MT can increase by several hundred times, thereby initiating melatonin signaling and enhancing plant resistance to these stresses [10,11,12]. Furthermore, it acts as a signaling molecule, regulating the expression of genes associated with the stress response, antioxidant enzyme production, and pathways of plant hormones [13]. Previous studies have shown that MT can upregulate the activity of the class A1 heat shock transcription factor (HSFA1s) and the production of the heat shock protein genes HSP90 and HSP101. This contributes to the enhancement of *Arabidopsis* heat resistance mediated by MT [13].

Enolases (2-phospho-D-glycerate hydratase; EC 4.2.1.11) are metalloenzymes [14]. It catalyzes the interconversion of 2-phosphoglycerate to phosphoenolpyruvate, which is the only dehydration step in the glycolytic pathway [15]. The enzyme has been extensively characterized in fungi, bacteria, animals, and plants, and plays an important role in biological fermentation metabolic pathways, especially glycolysis pathways [15]. The main structure of enolase is highly conserved; it consists of two identical subunits that are combined in a reverse-parallel manner. Each subunit has two domains, the N-terminal and C-terminal, and the N-terminal domain folds into a topology of β3α4 [16]. *Arabidopsis* has three enolase (ENO) isozymes, ENO1, ENO2, and ENO3/ENOc, which share 56–68% amino acid similarity, but ENO3/ENOc does not have enolase activity [17]. In plant studies, ENO1 has been less studied than ENO2, and most of these studies have focused on high salt stress [16]. Knockout of the AtENO2 gene in *Arabidopsis* was found to increase its susceptibility to salt stress. Consequently, the survival rate of mutant plants subjected to salt stress treatment was quite low [18]. The *AtENO2* gene exerts a negative regulatory effect on the expression of the *STZ/ZAT10* gene. It plays a crucial role in enhancing plant cold resistance by regulating the expression of *ZAT10* and other downstream genes, including *RD29A* [19]. Low-temperature stress enhances the expression of *TaENO-a* and *TaENO-b* genes in wheat [20]. Prior research has established that enolase *HSP48* in yeast functions as a heat shock protein, indicating that enolase also plays a significant role in heat resistance [21].

Researchers have extensively studied the biological role of enolase in plants; however, little information is available on how *ENO1* responds to heat stress. Therefore, the objectives of this study were to explore how MT enhances heat stress tolerance in maize, explore the regulatory mechanism of ENO1 against heat stress, and explore the mechanism by which MT responds to heat stress by regulating ENO1 gene expression. In the present study, we first quantitatively analyzed the expression site of the *ZmENO1 gene* and its expression pattern in response to heat stress, as well as after exogenous application of MT, by real-time fluorescence. Further, overexpressed maize lines and knockout lines of the *ZmENO1* gene were obtained by transgenic and gene editing techniques. The growth of wild-type plants (WT), overexpressed lines (OE), and knocked-out lines (*mutants*) of maize at the seedling stage were observed under normal (CK), heat stress (H), and heat stress +MT treatment (H+MT), and the molecular mechanism of MT regulating the *ZmENO1* gene in response to heat stress was elucidated at the physiological, biochemical, and molecular levels. The results of this study provide genetic resources for molecular design breeding and lay a solid foundation for further exploring the molecular mechanism of MT regulation of heat stress tolerance in maize.

## 2. Materials and Methods

### 2.1. Plant Material and Growth Conditions

The maize inbred line B104 was purchased from a company that produces genetically modified plants (Wimi Biotechnology Co., Ltd., Changzhou, China) in 2018. Seeds were germinated in a greenhouse at 28 °C under a 14-h light/10-h dark cycle for five days (5 d). At the three-leaf stage, plants were exposed to heat stress (42 °C) and melatonin (MT), 100 μmol/L treatment. Roots, young stems, and leaves were collected at the three-leaf stage; young stems, leaves, female ears, and male ears were collected at maturity to analyze the spatiotemporal expression pattern with three biological repeats. The samples were collected at different time intervals, including 0 h, 2 h, 4 h, 12 h, 24 h, 48 h, and 96 h, and immediately frozen in liquid nitrogen, and subsequently preserved at −80 °C.

### 2.2. RNA Extraction and RT-qPCR Analysis

Total RNA was extracted from the leaves of three distinct biological replicates at each time point. The first-strand cDNA was synthesized using a Hifair^®^ II 1st Strand cDNA Synthesis SuperMix (YEASEN, Shanghai, China). The gene-specific primers for qPCR were designed by Primer5 software, with reference to the corresponding sequence (Table S1). Actin 18s was used as an internal parameter. qPCR analyses were conducted using Hieff^®^ qPCR SYBR^®^ Green Master Mix (YEASEN, Shanghai, China) on a Light Cycler 480 instrument (Roche, Basel, Switzerland) following the guidelines provided by the manufacturer. The analysis was performed using three technical replicates per gene. The normalization of the relative expression level (2^−∆∆Ct^ 0 h) in the control plants that did not receive any treatment was set to 1. Quantitative real-time PCR (qRT-PCR) was utilized to quantify the *ZmENO1 gene*, antioxidant genes (*ZmPOD*, *ZmCAT*, *ZmAPX*, and *ZmSOD*), and osmotic potential response genes (*ZmP5CS* and *ZmProDH*).

### 2.3. Construction of Overexpression Lines of zmeno1

The cDNA of *ZmENO1* was used as a template, and the sequence-specific primers are listed in Table S1. The TaKaRa hi-fi enzyme (TAKARA, Dalian, China) was utilized to amplify the ORF gene. The recovered gel product was ligated to the T-vector, and the resulting plasmid was subsequently isolated. The positive monoclonal plasmid was subjected to double digestion with *Asc*I and *Bam*HI enzymes, along with the target vector (pFGC5941), resulting in the detection of the appropriate target fragment and vector through double digestion with T4 ligase, which was then transformed into *Escherichia coli* DH5α. A positive mono-clone of the targeted gene (*ZmENO1* connected to the target vector pFGC5941) was identified, and the *Agrobacterium tumefaciens* GV3101 receptor was transformed for further sequencing verification. The constructed overexpression vectors were sent to the company for genetic transformation of maize, and the overexpressed plants were constructed.

### 2.4. Construction of Knockout Plants of zmeno1

Construction of the CRISPR *ZmENO1*-Cas9 carrier pBUE411 and screening *ZmENO1* shear targets on http://www.genome.arizona.edu/crispr/CRISPRsearch.html. (accessed on 3 February 2021) The primers used are presented in Table S1. sgRNA was amplified, the PCR products were purified and recycled to construct the plasmid, and the reaction system was transformed into *E. coli* DH5α. The plasmid cloned by sequencing was extracted, and the constructed pBUE411- ENO1 plasmid vector was extracted and transformed into the *A. tumefaciens EHA105* transgene. After the mono-clone was selected, the bacterial solution was sent to the company for genetic transformation of maize. Plant resistance to the pBUE411 vector was screened for the BAR gene. First, the BAR gene was amplified for primary screening, and the transgenic mutant lines were amplified by PCR using genome sequence amplification primers. The amplified PCR products were sent to the company for sequencing, and the target sites were compared for mutations.

### 2.5. Determination of Melatonin and MDA Content, Fresh Weight, Leaf RWC, and Survival Rate

The number of seedlings of the wild type, overexpressed type, and mutant type under normal, heat (H), and heat+melatonin treatment (MH) for 3 days (3 d) was counted, and the survival rate was calculated. Melatonin content was determined by Wei et al. [22]. The fresh weight and relative water content of leaves (RWC) were measured by the weighing method. Malondialdehyde (MDA) content was measured by the thiobarbituric acid method [23].

### 2.6. Determination of SOD, POD, CAT, and APX Activities, H_2_O_2_, O^2−^, and GSH Content

The activity of peroxidase (POD) was assessed using the guaiacol method, while the activity of superoxide dismutase (SOD) was evaluated using the nitrogen blue tetrazole photochemical reduction method. The extraction of ascorbate peroxidase (APX) and catalase (CAT) was carried out using a 0.05 M phosphate buffer solution that contained 1% polyvinylpyrrolidone (PVP) with a pH of 7.5. The concentration of hydrogen peroxide (H_2_O_2_) was quantified using a ferrous oxidation-xylenol orange (FOX) assay. The NBT method was utilized to detect the presence of the superoxide anion O^2−^. The quantification of Glutathione (GSH) was carried out using a spectrophotometer. The experiment was conducted with three technical replicates. The enzyme extraction process involved the removal of seedlings from three distinct cups. Each cup produced a distinct copy. The activities of all enzymes were measured at 30 °C. Except for the enzymes, each component of the enzyme assay system began to react after being pre-incubated at 30 °C for 20 min.

### 2.7. Determination of the Rate of Electrolyte Leakage (REL), Water Potential, Pro and Soluble Protein Content

The number of seedlings of the wild type (WT), overexpressed type, and mutant type under normal, H, and MH treatments for 3 d were counted, and the third leaf was selected to determine the relative electrolyte leakage (REL) and water potential. The REL was measured according to the method of Wang Xuekui [24], and leaf water potential was measured with a WP4C dew-point water potential meter [25]. Free Proline was determined by ninhydrin colorimetry [24]. The SP content was determined by the Coomassie brilliant blue method [26].

## 3. Results

### 3.1. H and MH Treatments Induce Upregulation of ZmENO1 Expression

**Figure 1:**
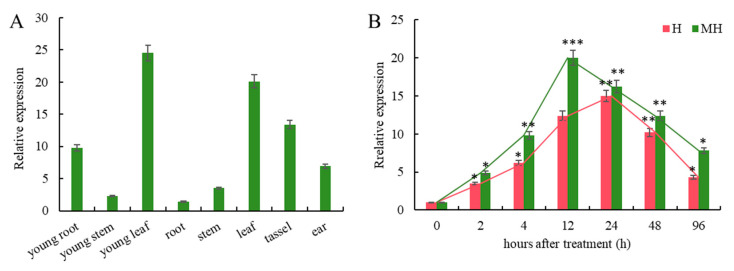
Heat stress and MT treatments induce upregulation of *ZmENO1* expression. (**A**) The expression of the *ZmENO1 gene* in the young roots, young stems, and young leaves of the maize trefoil stage and the roots, stems, leaves, male ear, and female ear of the maize mature stage. (**B**) Expression level of the *ZmENO1 gene* under heat stress (H) and stress with MT treatment (MH). Student’s *t*-test: *, **, and *** indicate a significant difference between each treatment and 0 h treatment at the 0.05, 0.01, and 0.001 levels, respectively.

To identify the expression levels of *ZmENO1* in various tissues of maize, we chose tissues from the three-leaf and mature stages, which included young roots, young stems, and young leaves; roots, stems, leaves, male tassel, and female ears. It was observed that the gene was expressed in all tested tissues. However, there were noticeable variations in the expression levels among different tissues and at different developmental stages. The expression level in young leaves was the highest, followed by that in mature leaves (Figure 1A). Moreover, we observed a significant increase in the expression level of *ZmENO1* within 2 h of exposure to heat stress, as well as with both heat stress and MT treatments in maize seedlings. The expression level of *ZmENO1* exhibited a sustained increase following exposure to H. It peaked at 24 h, experienced a 15-fold increase, and subsequently declined gradually. However, it consistently remained higher than that at 0 h (Figure 1B). Under MH treatment, the expression level of *zmeno1* was promptly enhanced, reaching its maximum level within 12 h, which was 20-fold higher compared to its expression before treatment. Ultimately, the level of expression steadily declined; however, it consistently remained higher than that at 0 h (Figure 1B).

### 3.2. MT Enhances Heat Tolerance in Maize Seedlings by UpregulatingZmENO1 Expression

**Figure 2:**
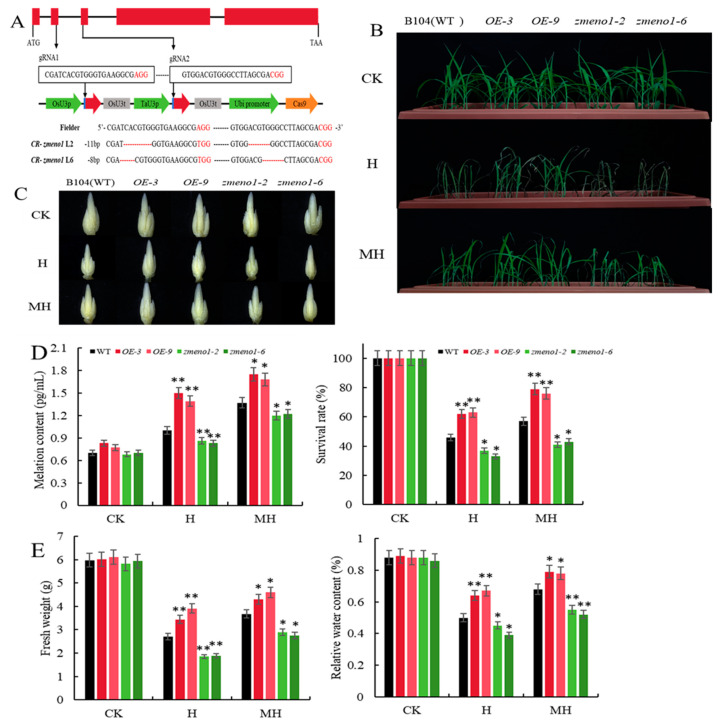
Melatonin enhances heat tolerance in maize seedlings by upregulating *ZmENO1* expression. (**A**) Target location and mutation sequence of *zmeno1* cleavage by Cas9. (**B**) Seedling phenotypes of WT, OE-3, OE-9, *zmeno1-2,* and *zmeno1-6* under CK, H, and MH treatments. (**C**) The developmental progress of tassels in WT, OE-3, OE-9, *zmeno1-2,* and *zmeno1-6* under CK, H, and MH treatments during the V9 stage. (**D**,**E**) Melatonin content, survival rate, fresh weight, and relative water content in the leaves of WT, OE-3, OE-9, *zmeno1-2,* and *zmeno1-6* plants (n = 3 ± SD). Student’s *t*-test: * and ** indicate significant differences between each treatment and 0 h treatment at 0.05 and 0.01 levels, respectively.

To explore the function of *ZmENO1* in H and determine whether MT influences the heat tolerance of *ZmENO1*, we generated overexpression lines by genetically transforming the maize inbred line B104. We selected two *ZmENO1-OE* lines (OE-3 and OE-9) with particularly high expression levels for the subsequent experiment (Figure S1). We obtained two knockout mutants (*zmeno1-2* and *zmeno1-6*) using CRISPR/Cas9-mediated genome editing of B104. Cleavage sites were identified within the second and third exons of *ZmENO1*. The zmeno1-2 mutant contained a 6-bp deletion and a 5-bp deletion, and the zmeno1-6 mutant contained a 3-bp deletion and a 5-bp deletion in the coding region of *ZmENO1*; both alleles resulted in a frameshift mutation (Figure 2A). Mutations that resulted in the deletion of translated amino acids and frameshift mutations were selected for further experimentation (Figure 2A). There were no significant differences in the growth of all plants under CK. However, when exposed to H, OE-3 and OE-9 plants exhibited superior growth compared to the WT, and the extent of leaf wilting was reduced. The *ZmENO1* mutants showed the poorest development and displayed severely wilted leaves. Under MH, all plants exhibited improved growth compared to H, but inferior growth compared to CK. However, ZmENO1-OE lines exhibited significantly superior growth compared to plants that were subjected to H, followed by WT (Figure 2B).

Furthermore, we assessed the developmental progress of maize tassels in WT and ZmENO1-OE plants at the V9 stage under both H and MH conditions. The results revealed that H caused a delay in the formation of maize tassels in all plants. Among them, the development of *zmeno1* mutants was the slowest (Figure 2C). These findings demonstrated that H had a significant impact on the growth of the tassel in *zmeno1* mutants. However, under MH treatment, the delay in the development of tassels was alleviated in all plants; the most prominent remission and fastest development was observed in ZmENO1-OE lines, while the development of *zmeno1* mutants showed the slowest progress (Figure 2C).

The results illustrate that the H and MH treatments significantly upregulated the endogenous melatonin content in all plants. However, the increase in MT in ZmENO1-OE lines (OE-3 and OE-9) was greater than that in WT, while the increase in WT was greater than that in *zmeno1-2* and *zmeno1-6* mutants (Figure 2D).

There were no significant differences in survival rate, fresh weight, or relative water content among all plants under CK (Figure 2E). However, under H, these indicators were highest in ZmENO1-OE lines, followed by WT and *zmeno1* mutants. Under MH treatments, the measured indicators of all plants exhibited greater values than those observed under H but lower than the values observed under CK. However, the indicators in ZmENO1-OE lines exhibited a greater increase compared to H, followed by WT (Figure 2E). These findings suggest that *ZmENO1* can enhance heat tolerance. Additionally, MT mitigated the harm caused by heat stress in maize plants. This mitigation effect may be attributed to the upregulation of *ZmENO1* expression level, which could potentially strengthen heat tolerance.

### 3.3. MT Upregulates ZmENO1 Expression to Enhance the Antioxidant Activities in Maize Seedlings

**Figure 3:**
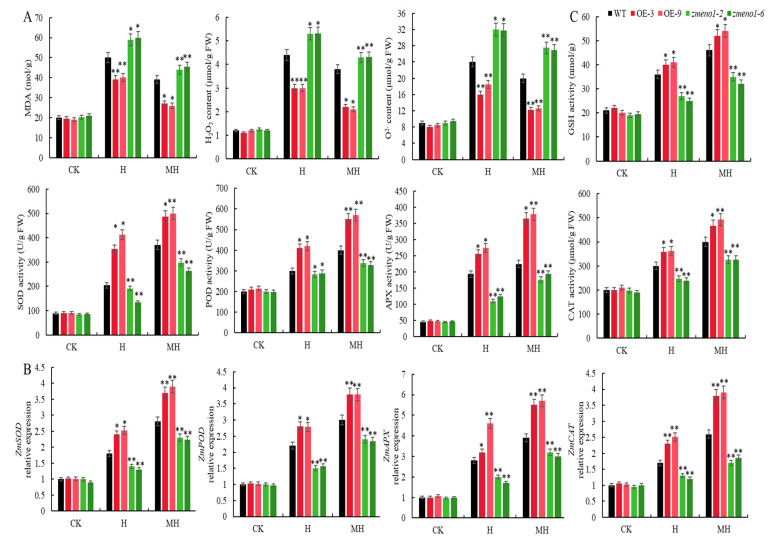
MT upregulates *ZmENO1* expression to enhance the antioxidant system in maize seedlings. (**A**) MDA, H_2_O_2_, and s O^2−^ contents of WT, OE-3, OE-9, *zmeno1-2,* and *zmeno1-6* under CK, H, and MH treatments (n = 3, ±SD). (**B**) The expression levels of *ZmSOD*, *ZmPOD*, *ZmAPX*, and *ZmCAT* in WT, OE-3, OE-9, *zmeno1-2,* and *zmeno1-6* under CK, H, and MH treatments (n = 3, ±SD). (**C**) GSH content and SOD, POD, APX, and CAT activities of WT, *Zm* WT, OE-3, OE-9, *zmeno1-2,* and *zmeno1-6* under CK, H, and MH treatments (n = 3, ±SD). Student’s *t*-test: * and ** indicate significant differences between each treatment and 0 h treatment at 0.05 and 0.01 levels, respectively.

The MDA content serves as a crucial physiological indicator in the assessment of leaf membrane impairment caused by heat-induced stress. Two important reactive ROS, hydrogen peroxide (H_2_O_2_) and superoxide anions (O^2−^), are produced by plants under stress. Under H, the MDA, H_2_O_2_, and O^2−^ contents increased significantly in all plants, except for ZmENO1-OE plants, compared to WT. Moreover, the MDA, H_2_O_2_, and O^2-^ contents in WT were significantly lower compared to those in the *zmeno1*-2 and *zmeno1*-6 mutants (Figure 3A). Under MH treatment, MDA, H_2_O_2_, and O^2−^ contents for all plants were significantly lower compared to those under heat stress treatment but greater values compared to those under CK conditions. However, MDA, H_2_O_2_, and O^2−^ contents of OE-3 and OE-9 plants were found to be significantly lower compared to those of H treatment followed by WT (Figure 3A). H affects the balance of ROS in maize cells and causes disruption to cell membranes, but ZmENO1-OE lines reduce the degree of damage to maize plants.

The antioxidant enzymes, including SOD, POD, APX, and CAT, along with GSH, form a circulating system that effectively scavenges free radicals. We found that the expression levels of *ZmSOD*, *ZmPOD*, *ZmAPX*, and *ZmCAT* in all plants significantly increased under H. The expression levels of these antioxidant enzymes in ZmENO1-OE lines were significantly higher than those in the WT. The expression levels in the WT were significantly greater than those in the *zmeno1* mutants. Under MH treatments, the expression levels of these antioxidant enzymes in all plants were higher than those under heat stress. However, the increase in expression levels in ZmENO1-OE lines was significantly higher than that in H treatment, followed by WT (Figure 3B). The pattern of antioxidant enzyme activity aligns with the changes in the expression levels of these genes. Under H treatment, the GSH content and SOD, POD, APX, and CAT activities of all plants significantly increased. However, these indicators in ZmENO1-OE plants were significantly higher than those in WT plants, and the activities of these indicators were significantly higher than those in the *zmeno1* mutants. Under MH treatments, these indicators for all plants were higher than those for the heat stress treatment. The increase in these indicators in ZmENO1-OE plants was significantly higher than that in the H treatment, followed by WT (Figure 3C).

Overall, these results suggest that ZmENO1-OE can augment the efficacy of antioxidant enzymes in maize seedlings, eliminate harmful substances, and thereby mitigate the harm caused by heat stress to cellular membranes. The exogenous application of MT improved the ability of maize to withstand heat stress by increasing the expression of *ZmENO1*.

### 3.4. MT Upregulates ZmENO1 Expression to Enhance the Content of Osmoregulatory Substances in Maize Seedlings

**Figure 4:**
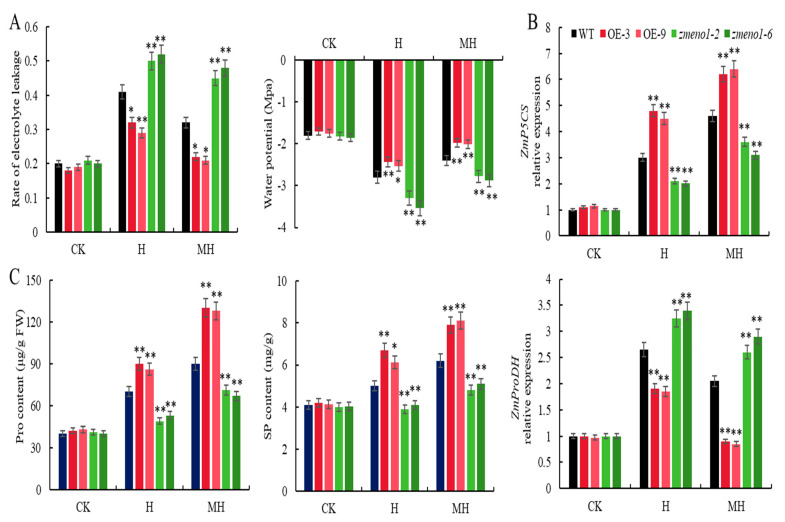
MT upregulates *ZmENO1* expression to enhance the content of osmoregulatory substances in maize seedlings. (**A**) The rate of electrolyte leakage (REL) and water potentials of WT, OE-3, OE-9, *zmeno1-2,* and *zmeno1-6* under normal, H, and MH treatments (n = 3, ±SD). (**B**) The expression levels of *ZmP5CS* and *ZmProDH* in WT, OE-3, OE-9, *zmeno1-2,* and *zmeno1-6* under normal, H, and MH treatments (n = 3, ±SD). (**C**) Pro and SP contents of WT, OE-3, OE-9, *zmeno1-2,* and *zmeno1-6* under normal (CK), H, and MH treatments (n = 3, ±SD). Student’s *t*-test: *, and ** indicate a significant difference between each treatment and 0 h treatment at 0.05, and 0.01 levels, respectively.

Under the CK treatment, the electrolyte leakage (REL) rate and water potential in all plants were consistently low, with no discernible variation among them (Figure 4A). Following heat stress, all plants exhibited an increase in both REL and water potential. Notably, *zmeno1* mutants displayed the highest REL and water potential, whereas ZmENO1-OE lines showed the lowest REL and water potential. Exogenous application of MT resulted in a decrease in REL in all plants, with the greatest drop observed in ZmENO1-OE lines and a moderate reduction in WT, while the *zmeno1* mutants maintained the highest REL and water potential (Figure 4A).

Under heat stress treatment, the expression levels of *ZmP5CS* and *ZmProDH* in all plants significantly increased. The expression levels of *ZmP5CS* and *ZmProDH* in ZmENO1-OE lines were significantly higher than those in WT, and the expression levels in WT were significantly higher than those in *zmeno1* mutants. Under MH treatment, these expression levels in all plants were higher than those under heat stress treatments. The increase in expression levels in ZmENO1-OE lines was significantly higher than that in heat stress treatment, followed by WT (Figure 4B).

The correlation between Pro and SP content was consistent with the changes in the expression levels of these genes. Under the heat stress treatment, the Pro and SP contents of all plants significantly increased. However, in ZmENO1-OE lines, the Pro and SP contents were significantly higher than those in WT, and the content in WT was significantly higher than that in *zmeno1* mutants. Moreover, under MH treatments, the Pro and SP contents of all plants were higher than those under heat stress treatments. However, the increase in Pro and SP content was significantly higher in ZmENO1-OE lines compared to that in the H treatment, followed by WT (Figure 4C).

We demonstrated that *ZmENO1* leads to an elevation in the levels of osmoregulatory substances in maize seedlings. Conversely, the maintenance of intracellular and extracellular osmotic potentials likely mitigates the detrimental effects of heat stress on cell membranes. Exogenous application of MT can increase the ability of maize to withstand heat stress by upregulating *ZmENO1* expression.

## 4. Discussion

Heat stress significantly affects crop quality and yield. Hence, it is imperative to develop new heat-resistant cultivars to mitigate the detrimental effects of heat stress in plants. Despite the acknowledged significance of enolase in plant stress response, research into the specific relationship between ENO1 and heat stress in maize is quite limited. The potential of MT to modulate *ENO1* expression levels in response to heat stress has not been previously reported, despite its role as a crucial growth regulator and antioxidant. In this study, *zmeno1* mutants and ZmENO1-OE lines were obtained by genome editing and transgenic technology. Moreover, we investigated the mechanisms by which melatonin improves heat tolerance in maize seedlings at the phenotypic, physiological, biochemical, and molecular levels. These results demonstrate that melatonin enhances heat tolerance by boosting the activity of antioxidant enzymes and osmotic regulatory substances. Additionally, the genetic results demonstrate that MT upregulates the expression level of *zmeno1* in maize seedlings.

### 4.1. MT Enhances Heat Tolerance in Maize Seedlings by Upregulating zmeno1 Expression Level

The accumulation and distribution of maize dry matter were regulated by photosynthetic features, and biotic and abiotic stresses. After heat stress, the chloroplast internal structure of the mesophyll cells of maize plants is damaged, and leaf greening and senescence are accelerated, resulting in a decrease in photosynthetic capacity. The cellular integrity of maize is compromised, resulting in disruption of its internal structure. This leads to a loss in the photosynthetic ability of leaves and a reduced ability to synthesize organic matter. Additionally, the plant’s ability to retain water was impaired, resulting in a decline in water content and fresh weight. Ultimately, these factors contribute to a decrease in the survival rate of the plant. Survival rate, relative water content, and fresh weight are direct indicators of the extent of damage to maize caused by heat stress. Previous studies have found that the application of exogenous MT significantly increased maize row number, ear diameter, ear length, 100 grain weight, and yield per plant after heat stress. Additionally, it has been observed that exogenous application of MT improves the rate of chlorophyll synthesis, thereby sustaining a high level of photosynthetic performance [27].

This study investigated the impact of heat stress on seedling and tassel development stages. By treating ZmENO1-OE lines and *zmeno1* mutants with heat stress and exogenous MT, it was found that heat stress significantly affected the growth of maize at both stages. However, the ZmENO1-OE lines showed less susceptibility to heat stress compared to the *zmeno1* mutants, which were the most severely affected (Figure 2B,C). Following MH treatment, the ZmENO1-OE lines showed the greatest increase in MT content, while the mutant lines showed the lowest increase in MT content (Figure 2D). The results showed that after exogenous application of MT under heat stress, the heat tolerance of all plants increased; the ZmENO1-OE lines showed the best growth and high survival rate, while the *zmeno1* mutants performed the poorest. Under heat stress, the relative water content and fresh weight of all plants decreased, but ZmENO1-OE lines showed a minor change in REL, and fresh weight and *zmeno1* mutants showed the largest decline. Compared with heat stress, the two indexes in ZmENO1-OE lines under MH treatment had the highest increase and the highest content (Figure 2E). The results illustrate that H and MH treatments induced the accumulation of MT and enhanced the heat tolerance of maize seedlings by upregulating the expression level of *ZmENO1*.

### 4.2. The Effect of Antioxidant Enzyme Activity on Plant Stress Tolerance

Heat stress not only impacts plant growth and development but also influences a range of physiological and biochemical processes. High temperature disrupts the equilibrium between the production and removal of reactive ROS and induces the accumulation of harmful substances such as MDA, H_2_O_2,_ and superoxide ions. This process triggers membrane lipid peroxidation and impairs the integrity of the cell membrane [28,29]. Plants under high-temperature stress do not simply endure the harm but adapt to the high-temperature adversity through active adjustment. Plants mitigate the extent of damage to cell membranes caused by lipid peroxidation by stimulating the activity of antioxidants and promoting the action of antioxidant oxidases [30,31].

MT has the ability to directly eliminate H_2_O_2_ or indirectly remove ROS by enhancing the activity of antioxidant enzymes [32]. These two systems work in synergy to enhance stomatal movement and, hence, increase drought tolerance [33]. Moreover, Melatonin frequently engages with other signaling molecules to effectively control the redox equilibrium in plants and enhance their ability to tolerate high levels of salt [34]. MT can improve the activity of L-Dcysteine desulfhydrase (L-/DCD), induce the production of H_2_S, regulate the expression of the antioxidant enzyme gene, improve the activity of the antioxidant enzyme, and alleviate the ROS outbreak under salt stress [35]. MT, as an antioxidant, can remove excess ROS produced under salt stress and can also remove ROS by regulating the transcription level of related factors in the antioxidant system and improving the activities of the antioxidant enzymes CAT, POD, and SOD [36]. These help to maintain the stability and integrity of cell membranes, mitigate cell membrane damage, and recover from salt stress [33]. Previous studies have demonstrated that MT can increase the activity of class A1 heat shock factor (HSFA1s) and the production of the heat shock protein genes HSP90 and HSP101. This contributes to the enhancement of Arabidopsis heat resistance mediated by MT [13]. Xu Chenxiao et al. found that MT has the ability to increase the expression of HSF7, HSP70.1, and HSP70.11 in Cucumis sativus seedlings. As a result, this reduces the accumulation of ROS and minimizes the damage caused by heat stress through peroxidation [37]. MT can enhance pepper tolerance to heavy metals by activating its antioxidant system and increasing the expression of cycling-related genes in the ascorbate-glutathione pathway (AsA-GSH) [38].

Our research demonstrated that heat stress led to an increase in the levels of MDA, H_2_O_2_, and O^2−^ in all plants. However, after applying the MT spray, the levels of these three indicators decreased in all plants. This decrease was particularly pronounced in ZmENO1-OE plants, in which the levels of MDA and ROS were the lowest. Conversely, *zmeno1* mutants exhibited the highest levels of ROS (Figure 3A). Heat stress increased the expression levels of *ZmSOD*, *ZmPOD*, *ZmAPX*, and *ZmCAT* in all lines. However, the ZmENO1-OE lines exhibited the greatest increase in expression levels following MT spraying, while the *zmeno1* mutants showed the lowest increase (Figure 3B). These results suggest that upregulation of *ZmENO1* can enhance the activity of antioxidant enzymes in maize seedlings, eliminate harmful substances, and thus mitigate the harm caused by heat stress to cell membranes (Figure 3C). Furthermore, the application of MT can induce the upregulation of *ZmENO1*, and the addition of exogenous MT can improve the ability of maize to withstand high temperatures by increasing the expression of *ZmENO1*.

### 4.3. The Effect of Content of Osmoregulatory Substances on Plant Stress Tolerance

Plants enhance their ability to withstand high temperatures by utilizing not only the antioxidant enzyme system but also osmoregulatory chemicals, including Pro, SP, and betaine. Multiple studies have demonstrated that Pro and SP, which are commonly found in osmoregulatory substances, accumulate in significant amounts during periods of stress. This accumulation serves to stabilize the protoplasmic colloid and metabolic processes in tissues, prevent cytoplasmic dehydration, and safeguard the integrity of the plasma membrane [39,40,41]. In *Capsicum annuum* L., the application of exogenous MT enhances the levels of proline during the seedling and flowering stages. This helps to regulate cell osmotic pressure, decrease membrane peroxidation, and ultimately mitigate the harmful effects of cold stress [42]. Exogenous MT can significantly reduce the REL of *Beta vulgaris* L., increase the SP and free Pro levels, and maintain cell homeostasis [43].

Our results indicate that heat stress led to elevated levels of REL and water potential in all lines. However, spraying with MT resulted in a reduction in these levels in all lines, particularly in ZmENO1-OE lines with the highest and lowest levels, as well as in *Zmneo1* mutants with the highest levels (Figure 4A). Heat stress increased the expression levels of *ZmP5CS* and *ZmProDH* in all lines, with the highest upregulation observed in ZmENO1-OE plants, followed by the WT (Figure 4B). However, after spraying MT, the gene expression in all lines increased, and the increase in gene expression level was the largest and highest in the ZmENO1-OE lines and the lowest in the *Zmneo1* mutants (Figure 4B). These changing trends in Pro and SP content were consistent with the changing trend in the expression of these genes (Figure 4C). These results together suggest that ZmENO1-OE can increase the content of osmoregulatory substances in maize seedlings, maintain intracellular and extracellular osmotic potential, and thereby reduce the damage caused by heat stress to cell membranes. Exogenous application of MT can enhance the resistance of maize to heat stress by upregulating *ZmENO1* expression.

**Figure 5:**
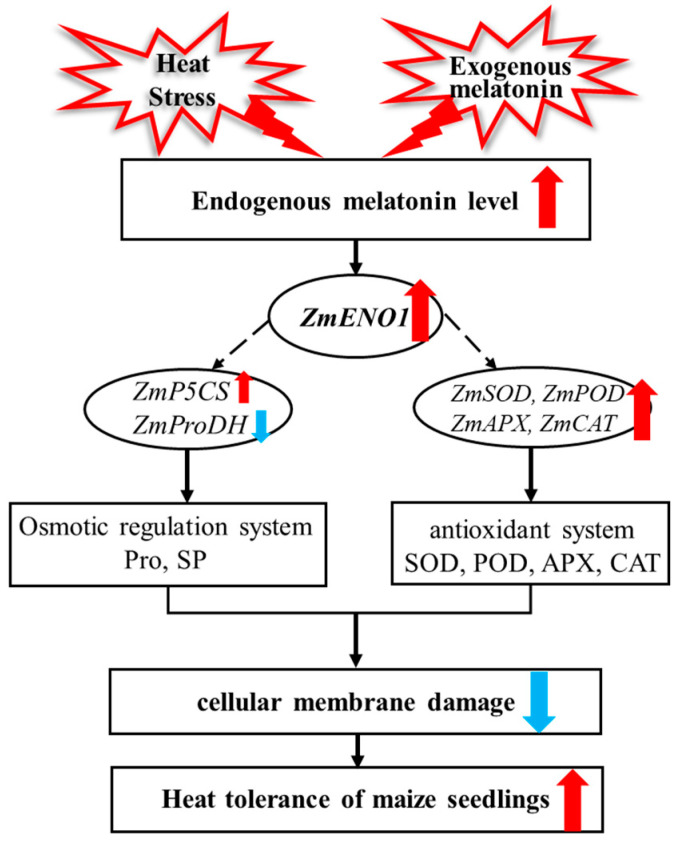
Working models showing the *ZmENO1* regulatory pathway activated in response to heat stress and exogenous melatonin. Blue arrows represent a decrease, while red arrows represent an increase.

We conclude that heat stress and exogenous application of MT can induce the accumulation of endogenous MT. The accumulated MT can upregulate the expression level of *ZmENO1*. Through the above analysis, it becomes apparent that there are two ways for *ZmENO1* to regulate heat stress, including the upregulation of genes related to antioxidant enzyme activity (*ZmSOD*, *ZmPOD*, *ZmAPX*, and *ZmCAT*) and osmoregulatory substances (*ZmP5CS* and *ZmProDH*). Furthermore, it enhances the activity of antioxidant enzymes and the content of osmoregulatory substances, reduces the damage caused by heat to cell membranes, and thus enhances the heat tolerance of maize under heat stress (Figure 5).

Horizontal lines with arrows represent a boost in gene expression levels or heat resistance physiological indicators. Discontinuous horizontal lines with arrows represent the direct or indirect promotion of gene expression levels. The red arrow indicates promotion, and the blue arrow indicates inhibition.

Interestingly, H_2_O_2_ has been identified as a key element in the MT-induced regulation of stress responses, with previous studies reporting that MT maintains low H_2_O_2_ levels either directly by binding to H_2_O_2_ or indirectly by upregulating antioxidant enzyme levels [44,45]. Our findings showed that MT effectively reduced the levels of H_2_O_2_, which were generally elevated during heat stress (Figure 3A). Additionally, MT significantly increased the activities of SOD, APX, GSH, CAT, and POD under heat stress (Figure 3C). Collectively, these findings indicate that MT’s initial mechanism of action in assisting plants in delaying or preventing heat stress reactions is by regulating H_2_O_2_ levels. Previous studies have demonstrated that plants significantly elevate the concentrations of osmotic chemicals in order to mitigate cell harm caused by unfavorable environmental circumstances [46]. Our research reveals that MT reduced the heat-induced increase in Pro and SP levels, leading to improved heat tolerance in maize seedlings (Figure 4).

Previous studies have confirmed that MT can upregulate or inhibit the expression of some genes, thereby regulating plant stress resistance. Numerous studies have demonstrated that MT induces class A1 heat shock factors (HSFA1) that are possibly involved in heat tolerance in *Arabidopsis* [13]. MT improves the ability of banana fruit to withstand low temperatures and reduces the occurrence of peel browning during storage [47], and MT affects the photosynthetic performance of pepper seedlings under cold stress [43]. Melatonin has been found to enhance drought tolerance by affecting jasmonic acid and lignin biosynthesis in wheat (*Triticum aestivum* L.) [48]. Impact of exogenous MT application on photosynthetic machinery under abiotic stress conditions [49]. However, the genes involved in these studies have not been verified. Further, it is necessary to conduct functional studies on the selected genes to determine their role in MT-induced stress resistance. In our study, we confirmed that *zmeno1* was upregulated by heat stress and exogenous application of MT and also confirmed their involvement in MT-enhanced heat tolerance through ZmENO1-OE lines and *zmeno1* mutants. However, additional investigation is necessary to examine the effects of heat stress in field conditions in order to establish MT’s potential as a chemical agent for enhancing heat tolerance in crops.

## 5. Conclusions

To summarize, our results demonstrate that *ZmENO1* is upregulated in response to heat stress and exogenous application of MT. *ZmENO1* enhances heat tolerance in maize seedlings. MT can increase the activity of antioxidant enzymes and the accumulation of osmoregulatory chemicals by upregulating the expression of *ZmENO1*. This, in turn, reduces the harm caused by high temperatures to cell membranes and eventually enhances the heat tolerance of maize seedlings.

## Data Availability

The data presented in this study are available upon request from the corresponding author.

## Supplementary Materials

The following supporting information can be downloaded at: https://www.mdpi.com/article/10.3390/antiox13091144/s1, Figure S1: Expression levels of *ZmENO1* overexpressed transgenic lines were detected. Table S1: List of primers used in present study.
