# Supplementary material for: Melatonin Enhances Heat Tolerance via Increasing Antioxidant Enzyme Activities and Osmotic Regulatory Substances by Upregulating zmeno1 Expression in Maize (Zea mays L.)
Source: Antioxidants (Basel). 2024 Sep 22;13(9):1144. doi: 10.3390/antiox13091144 (PMC11429225; doi:10.3390/antiox13091144)
Supplement: Supplementary file 1 [file antioxidants-13-01144-s001.zip › antioxidants-3137669-supplementary.pdf]

Supplementary Table and Figures

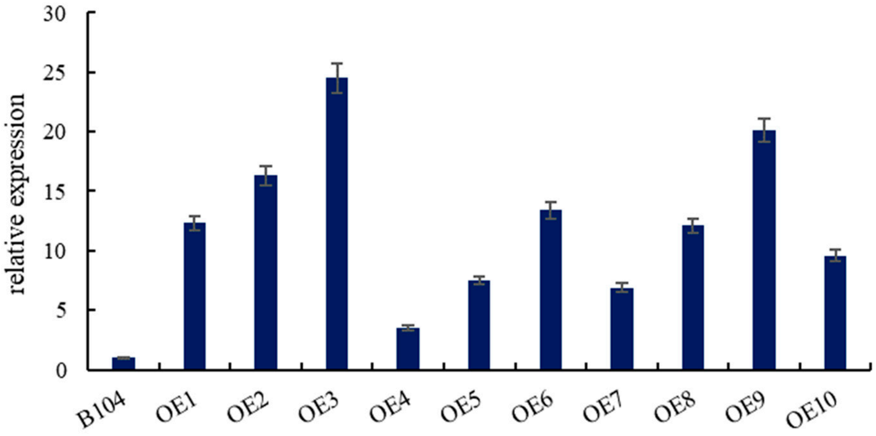

Figure S1. Expression levels of *ZmENO1* overexpressed transgenic lines were detected.

Table S1. List of primers used in present study.

Primer names	Primer sequences (5'-3')	Function
ZmENO1-F1	AATAATGGTCTCAGGCGCGATCACGTGGGTGAAGGCG	Construction of knockout vector
ZmENO1-F2	GCGATCACGTGGGTGAAGGCGGTTTTAGAGCTAGAAATAGC	Construction of knockout vector
ZmENO1-R1	AACTCGCTAAGGCCCACGTCCACGCTTCTTGGTGCC	Construction of knockout vector
ZmENO1-R2	ATTATTGGTCTCTAAACTCGCTAAGGCCCACGTCCAC	Construction of knockout vector
ZmENO1-AscI-F	TTGGCGCGCCATGGCGGTCACGATCAC	Construction of overexpression vector
ZmENO1-BamH I-R	CGGGATCCTTAGTAGGGCTCCACTGGT	Construction of overexpression vector
ZmSOD-F	AGTTCGGTTCTGGATGGGTT	Detection of qRT-PCR
ZmSOD-R	GGGTTGATGGCATTTGGAG	Detection of qRT-PCR
ZmPOD-F	GCCACTTTCCTTCCGAGTCATA	Detection of qRT-PCR
ZmPOD-R	CACTGTAATAAGGCCGGTTGTG	Detection of qRT-PCR
ZmCAT-F	CAGGCTGTCGTGAGAAGTGC	Detection of qRT-PCR
ZmCAT-R	AGATCCAAATGGTACGGTGTTC	Detection of qRT-PCR
ZmAPX-F	TACGCCGATTTCTACCAGCT	Detection of qRT-PCR
ZmAPX-R	GCAACAATGTCCTGATCGCT	Detection of qRT-PCR
ZmP5CS-F	GCAAGTTGATAGTGCCGCTGTGTT	Detection of qRT-PCR
ZmP5CS-R	ACTCCCTTGTCACCATTCACCACT	Detection of qRT-PCR
ZmProDH-F	GCAACCGTGTTCTTCTCTCTC	Detection of qRT-PCR
ZmProDH-R	CAAGCGAGGGGAACGTAGAG	Detection of qRT-PCR
18S-F	CCTGCGGCTTAATTGACTC	Detection of qRT-PCR
18S-R	GTTAGCAGGCTGAGGTCTGG	Detection of qRT-PCR
